# The Origin and Evolution of Bladder Cancer Stem Cells

**DOI:** 10.3389/fcell.2022.950241

**Published:** 2022-07-12

**Authors:** Jiufeng Tan, Yao Wang, Lihui Sun, Siqi Xu, Charles Li, Xuefei Jin

**Affiliations:** ^1^ 2nd Inpatient Area of Urology Department, China-Japan Union Hospital of Jilin University, Changchun, China; ^2^ Key Laboratory of Urology Tumor of Jilin Province, Changchun, China; ^3^ Zhongke Jianlan Medical Research Institute, Beijing, China

**Keywords:** bladder cancer, cancer stem cells, biomarkers, signaling pathway, targeted therapy

## Abstract

Bladder cancer is the most common malignant tumor of the urinary system. Bladder cancer stem cells (BCSCs) play key roles in tumor initiation, metastasis, relapse and drug-resistance. Investigation of BCSCs is of great value. On the basis of a review of normal bladder stem cells and universal cancer stem cells (CSCs), we summarize the origin of BCSCs, isolation and identification of CSCs from bladder cancer, signaling pathway of BCSCs, BCSCs targeted therapy, and relationship of BCSCs with non-muscle invasiveness and muscle invasiveness. This review aims to provide better elucidation about BCSCs, and provide constructive data for classification, prognosis, treatment and early intervention of bladder cancer.

## Introduction

Cancer stem cells (CSCs) play an important role in tumor survival, proliferation, metastasis and recurrence. CSCs maintain the vitality of tumor cell populations through self-renewal and unlimited proliferation; the movement and migration capabilities of CSCs make tumor cell metastasis possible. These functions are considered to give rise to formation of heterogeneous tissue and high recurrence rates of tumor (Easwaran et al., 2014). At the same time, there were evidence showed that CSCs present in many malignancies, including bladder cancer.

Bladder cancer is one of the major global problems that need imperative solutions. It has typical clinical features such as high recurrence rate, high metastasis rate and prominent heterogeneity. In academia, it is generally believed that bladder cancer CSCs are most likely to be the original causes of clinical features and other complex biological behaviors of bladder cancer. Therefore, it is important to further study and reveal the mechanisms of CSCs in tumor behaviors such as bladder cancer recurrence and metastasis. This poses practical significance by providing possible new research directions and therapeutic targets as measures against high recurrence rate and high metastasis rate of bladder cancer.

## Bladder Stem Cell and Bladder Epithelial Differentiation

Bladder epithelium stores urine and provides a protective barrier to bladder from urine. Bladder epithelium can be divided into three cell layers based on degree of differentiation: basal layer, middle layer and umbrella cell layer. Basal layer is located between urothelial cells and lamina propria, composed of undifferentiated small cubic cells; umbrella cell layer is a large, flat cell layer on the surface; middle layer is composed of cuboid cells with sizes between basal cells and umbrella cells. When bladder mucosa is damaged by pathogens (usually by bacterial infection), stem cells in urothelium which are initially at rest phase can resume proliferation and provide repair for the damaged epithelium (Shin et al., 2011).

Epithelial integrity of organ is maintained by regulation of proliferation and differentiation of stem cells. Although epithelium of certain organs (such as intestine) maintain the ability to regenerate and proliferate continuously, mammalian bladder can be transformed from an almost static state to a highly proliferative state under induction of epithelial damage. The mechanism of damage-induced regenerative response remains unclear. Shin et al. revealed that Hedgehog/Wnt feedback loop enhanced bladder epithelial stem cells regeneration and proliferation. Proliferative response induced by infection or injury of bladder was regulated by the signal feedback between urothelial basal cells and stromal cells located in underneath layer (Shin et al., 2011). Urothelial basal cells could regenerate into all types of urothelial cells. In response to injury, expression of protein signal Sonic hedgehog (Shh) in basal cells increased and this elicited increased expression of matrix Wnt signaling protein, and ultimately proliferation of urothelium and stromal cells was stimulated via feedback. Shin et al. believed that enhancement of signal feedback loop activities and increased proliferation of related cells might be necessary for recovery of urothelial function. Besides, it could help clearing pathogens and prevent further spread of infection in case of bacterial damage of epithelium. This study provided a conceptual framework for damage-induced epithelial regeneration of endoderm organs and an important experimental basis for understanding of signaling pathways in cancer growth and metastasis.

However, specific location of bladder stem cells remains controversial. It is conventionally believed that undifferentiated urothelial cells are precursor cells of two other bladder epithelial cells; umbrella cells are the most differentiated bladder epithelial cells; and basal cell layer provides a perfect survival environment for stem cells, protecting stem cells from damage by toxic substances. Therefore, most researchers tend to believe that bladder stem cells are located in basal cell layer (Chan et al., 2009). In 2008, Kurzrock and his colleagues identified a type of cells with slow renewal in mouse bladder and they were presumed to be urothelial stem cells (Kurzrock et al., 2008). These cells are called “label-retained cells”, they can retain BrdU-labeled DNA for a longer period of time, indicating that these cells are proliferating and renewing slower. In addition, labeled cells in basal layer appear to have significantly greater ability to form clones and proliferate than other cells, these are important properties of stem cells. Bladder stem cells can generate various types of urothelial cells, but this multidirectional differentiation is strictly regulated and controlled under normal conditions to prevent formation of tumor (Chan et al., 2009). It should be noted that, stem cells or more differentiated progenitor cells may cause tumors attributed to changes in genetics or epigenetics. Recent studies have found that some bladder cancers are derived from specific genetic or epigenetic changes, and that they correspond to type of clinical pathology (McConkey et al., 2010).

## Relationship Between BCSCs and Non-Muscle Invasiveness and Muscle Invasiveness

Bladder cancer can give rise to two different types of tumors, one is non-muscle invasive bladder cancer and the other is muscle invasive bladder cancer, via two different pathways (Dinney et al., 2004). The former is often accompanied by Ha-ras/FGFR3 gene mutation activation, which causes tumor to grow toward bladder cavity in the form of papillary lesions. Therefore, recurrence rate of this type of bladder cancer is as high as 70%. The latter is often accompanied by *p53/Rb/PTEN* gene inactivation (Cordon-Cardo, 2008). This type of bladder cancer has high proliferation rate and strong invasiveness, with about 50% of them can result in fatal metastasis. In academia, it is generally believed that CSCs initiate tumor formation; this is manifested in bladder cancer. Normal stem cells with Ha-ras or FGFR3 mutations may transform into CSCs leading to superficial non-muscle invasive bladder cancer. Stem cells with mutations in the p53/Rb/PTEN gene may become CSCs leading to muscle-invasive bladder cancer. In other words, high recurrence rate of papillary non-muscle invasive bladder cancer, high proliferation rate and strong invasiveness in muscle invasive bladder cancer are likely to be attributed to their corresponding CSCs; this theory is derived from studies using transgenic mice. In mice, continuous activation of the Ha-ras gene could cause non-invasive bladder cancer, while introduction of UP II promoter to SV40T gene functionally inhibited expression of p53 and pRb genes and caused development of invasive bladder cancer in experimental mice (Zhang et al., 2001; Cordon-Cardo, 2008). Primordial cells containing the above transgenes are highly likely to transform into bladder cancer CSCs, which in turn give rise to corresponding types of bladder cancer.

Fifteen percent of non-muscle invasive bladder cancers progress to muscle invasive bladder cancer after inactivation of the p53/pRB pathway (Wu, 2009). This finding provides strong support for hypothesis which denotes that p53/pRb inactivation promotes survival or proliferation of bladder cancer CSCs and increases metastasis potential of bladder cancer.

## Origin of Bladder Cancer CSCs

As discussed above, “CSCs” may be derived from normal stem cells that acquire proliferative advantage following mutations (Jordan, 2009). However, it has not been conclusive whether CSCs are derived from normal stem cells, progenitor cells, dedifferentiated cells, normal tumor cells or fusion cells (Bjerkvig et al., 2005). Yang et al. applied single-cell exome sequencing of a total of 59 cells from bladder cancer stem cells (BCSCs), bladder cancer non-stem cells (BCNSCs), bladder epithelial stem cells (BESCs), and bladder epithelial non-stem cells (BENSCs) (Yang et al., 2017). It was found that BCSCs were derived from BESCs or BCNSCs, which exhibited clonal homogeneity. Twenty-one driver mutations were identified in BCSCs, six of which had never been reported in bladder cancer (ETS1, GPRC5A, MKL1, AWR, PITX2, and RGS9BP). Generation of concurrent mutations in ARID1A, GPRC5A, and MLL2 significantly strengthened the self-renewal and tumor-initiating capabilities of BCNSCs. This study summarize the genetic basis of human BCSCs and it revealed origin of human BCSCs through evolutionary analysis.

There are four types of CSCs markers in bladder cancer, including CD44^+^, EMA-, 67LR, BCMab1+; they are located in basal cell layer of bladder tumor. This phenomenon has led to increasing speculation about origin of BCSCs. BCSCs may be derived from normal stem cells with altered epigenetics, if all markers are derived from a specific type of cells in bladder cancer. On the other hand, if these markers are derived from different types of cells, it can be assumed that BCSCs are derived from different progenitor or dedifferentiated cells, ultimately resulting in different subpopulations of BCSCs. Li et al. combined the uses of human bladder cancer cell monoclonal antibody BCMab1 and anti-CD44 antibody which specifically recognize bladder cancer marker AG-α3β1, and successfully isolated a subgroup of cells with stem cell characteristics (BCMab1+/CD44+ subgroup). This subpopulation of cells were proven to have strong proliferation and self-renewal ability (Li et al., 2016).

## Isolation and Identification of CSCs From Bladder Cancer

Cell surface markers are commonly used as a routine method for the isolation and identification of bladder cancer stem cells. Considering that tumor cell lines undergo long-term *in vitro* culture, their biological behaviors and antigenic phenotypes have changed. Therefore, primary cells or *in vivo* early passage cells may be the ideal models for studies of isolation and identification of bladder cancer CSCs.

Several experiments demonstrated that CD44^+^ bladder cells are 10–200 times more likely to give rise to tumor than CD44^−^ cells in immunodeficient mice, demonstrating their high tumorigenicity. Further, they remain able to demonstrate heterogeneity of primary tumor following serial passage transplantation, this fulfills the functional criteria of stem cells identification (Chan et al., 2009). In an experiment which assessed stemness of tumor cells using *in vitro* clonal formation assay, a subtype of CD44v6+ epithelial membrane antigen-negative (EMA-) BCSCs were isolated by using CD44 splice isoform (CD44v6). Another experiment successfully isolated 67LR + CEACAM- BCSCs using the 67L-kDa laminin receptor and carcinoembryonic antigen-associated cell adhesion factor 6 (CEACAM6). (Edris et al., 2012). In the above experiments, subsets of stem cells with tumor characteristics were isolated by using cell surface markers which are normally expressed in urothelial basal cells. BCSCs specific markers were listed in Table 1.

**TABLE 1:** BCSCs specific markers.

Markers	BCSCs	BCNSCs
CD44	CD44^+^	CD44^−^
CD133	CD133^+^	CD133^-^
CD47	CD47^+^	CD47^−^
ALDH	ALDH1^+^	ALDH1^-^
CK	CD44^+^/CK5^+^/CK20^-^	CD44^-^/CK5^-^/CK20^+^
OCT-4	OCT-4^+^	OCT-4^-^
Nanog	Nanog^+^	Nanog^-^
Bmi-1	Bmi-1^+^	Bmi-1^-^
ABCG2	ABCG2^+^	ABCG2^-^
MAGE-A3	MAGE-A3^+^	MAGE-A3^-^
BCMab1	BCMab1^+^/CD44^+^	BCMab1^-^/CD44^-^
Hoechst 33342	Hoechst 33342^+^	Hoechst 33342^-^

Another desirable BCSCs marker is ALDH1A1. Through *in vitro* small sphere formation assay and serial tumor transplantation using ALDH1A1 negative group as control, positive group was demonstrated to possess greater colony-forming ability and tumorigenicity. After shRNA was used to functionally knock down the *ALDH1A1* gene, clonality and tumorigenicity of bladder cancer CSCs were reduced. More interestingly, ALDH1A1+ cells were found to be a subtype of CD44^+^ cells, suggesting that such cells may be the more primitive bladder cancer CSCs (Su et al., 2010).

Isolation and identification of BCSCs can also be performed through the biological behavior of tumor cells. It is known that stem cells highly express ATP-binding cassette transporter, which allows them to actively pump drugs out of cells. Utilizing the dye-repellent property of the DNA fluorescent dye Hoechst 33,342 of CSCs, bladder cancer stem cells, also known as SP cells, can be isolated by flow cytometry; these cells have CSCs properties such as colony-forming, self-renewing, and multipotent as compared with non-SP cells (Ning et al., 2009).

The above experiments confirmed that functional BCSCs exist in primitive bladder cancer specimen, transplantation tumor and immortal bladder cancer cell line. With the advent of new methods for the isolation and identification of CSCs, it seems increasingly important to elucidate the relationship between bladder cancer heterogeneity and CSCs subtypes.

## Related Molecular Mechanism of CSC

Cancer stem cells have similar self-renewal capacity to normal stem cells. Signal pathways such as Wnt, Notch, and Hedgehog which are related to stemness maintenance and stem cells plasticity regulation are involved in stemness regulation of CSCs. In addition, RTKs signaling, IL-6 and TK1 signaling play important roles in regulating the stemness of solid CSCs.

### Hedgehog Signaling Pathway

The Hedgehog signaling pathway plays an critical regulatory role in embryogenesis and development. Among the three homologous genes of the Hedgehog gene (SHH, IHH and DHH), of which SHH has the highest expression (Hebrok, 2003). Hedgehog binds as a ligand to the transmembrane protein receptor Patched 1 (PTCH), which activates the GLI family of transcription factors and PTCH by releasing its inhibitory effect on Smoothened (Smo). A study found that abnormal activation of this pathway is involved in occurrence of a variety of tumors, and is associated with chemotherapy resistance of a variety of tumor cells. Blocking any molecular event of this pathway may weaken CSCs and thereby introducing benefits to patients (Monteiro and Fodde, 2010).

### Notch Signaling Pathway

Notch signaling affects multiple processes in normal cell morphogenesis, including differentiation of pluripotent progenitors, apoptosis and cell proliferation. This pathway includes four receptors (Notch1 to Notch4) and five ligands, and involves in expression regulation of multiple target genes (Wang et al., 2008). Through the interaction of Notch ligands of adjacent cells and receptors, Notch protein undergoes three cleavage, is released from the intracellular segment (NICD) into the cytoplasm, and enters the nucleus to bind to the transcription factor CSL to form a NICD/CSL transcription activation complex, so that the downstream target genes play a biological role. Studies found that Notch signaling pathway is over-activated in a variety of tumors and it plays an important role in maintaining stemness of CSCs (Pannuti et al., 2010). For examples, in melanoma, Notch4 interacts with CSCs expressing CD133 and ABCG2 and this promotes CSCs to form melanoma. Abnormal expression of Notch1, Hes1 and PTEN genes in invasive bladder cancer. Activation of Notch signaling pathway caused by abnormally high expression of Notch1 is one of the molecular events in invasive bladder cancer.

### Wnt Signaling Pathway

The Wnt signaling pathway is a complex network of proteins that function most commonly in embryonic development and cancer. This pathway is closely related to maintenance of stemness regulation in CSCs (Klaus and Birchmeier, 2008). The Wnt signaling pathway is a signal transduction pathway of a group of multiple downstream channels stimulated by the binding of the ligand protein Wnt and membrane protein receptors. Under normal conditions, Wnt signaling maintains stability of stem cell pool and prevents cell differentiation by stabilizing level of β-catenin in cytoplasm. Through this pathway, extracellular signals are transmitted into cells through the activation of intracellular segments of cell surface receptors.

Following activation of the Wnt signaling pathway, Wnt binds to cell surface receptors FZD and LRP5/6, thereby triggering intracellular signaling. First, cytosolic protein Dsh is activated and the activated Dsh then combines with Axin and Frat1. Under resting conditions, Axin forms a complex with GSK-3β and APC, Frat1 mediates detachment of GSK-3β from Axin and this inhibits phosphorylation of β -catenin. After the non-phosphorylated β-catenin accumulates to a certain extent in cells, it is transported to the nucleus to activate target genes. Target genes of β-catenin include protooncogene c-myc, matrix lyase, fibroblast growth factor, epithelial cell growth factor and cyclin D1. The expression products of these genes can promote tumor development. Therefore, when tumorigenic β-catenin mutations present, they may lead to dysregulation of Wnt pathway in CSCs and thereby induce tumorigenic proliferation (Teng et al., 2010).

### Other Signaling Pathway

Gene mutations activate the JAK-STAT signaling pathway and induce tumorigenesis. Studies have shown that the tumorigenicity of various CSCs is closely related to the activation of STAT3. In addition, IL-6 plays an important role in maintaining stemness of stem cells, and can allow conversion of non-CSCs into CSCs by regulating CSCs-associated OCT2, CD44 and SOX2 genes. Other studies have shown that the IL6/JAK/STAT3 signaling pathway maintains not only the plasticity of CSCs, but also stemness by activating the mTORC1-STAT3 signaling pathway. (Zhou et al., 2007; Wang et al., 2008). Tumorigenic tyrosine kinase receptor also plays an important role in stemness maintenance and chemoresistance of cancer stem cells (Singh et al., 2015).

## Resistance Mechanism of Bladder Cancer CSCs

The high proliferative, invasive and metastatic capacity of bladder cancer may be associated with BCSCs. Resistance of bladder cancer to chemotherapy and radiotherapy is also commonly thought to be due to BCSCs. On this basis, it is important to investigate drug resistance mechanisms of BCSCs (Tharakan et al., 2010). Drug resistance mechanisms of bladder cancer CSCs include: 1) active efflux of chemotherapeutic drugs; 2) enzymatic reactions inactivating drugs and their metabolites such as oxidants and free radicals; 3) inhibition of programmed cell death (apoptosis); 4) regulation of cytokines and other immune substances and subsequent automatic inhibition of anti-tumor immune responses.

### ABC Transporter

ABC transporters in stem cells, especially verapamil-sensitive ABCG2 proteins, are capable of pumping intracellular metabolites, drugs, and toxic substances out of cells. This serves as the basis for CSCs isolation by SP sorting method described previously (Schinkel and Jonker, 2003). Investigators isolated SP cells from T24 human bladder cancer cell line. Following culture, they confirmed that these SP cells demonstrated strong colony-forming ability and were resistant to chemoradiotherapy, indicating ABC transporter-mediated drug efflux plays a role in drug mechanism of bladder cancer (Ning et al., 2009).

### Acetaldehyde Dehydrogenase

Acetaldehyde dehydrogenase (ALDH) is highly expressed in BCSCs (He et al., 2009). In a transplantation mouse colon cancer model, ALDH enzymatic reactions oxidized and inactivated biological activities of cyclophosphamide metabolites, thereby allowed continued growth of tumor during treatment. These results suggest that inactivation of drug metabolites by ALDH high expression is likely to be one of the drug resistance mechanisms in bladder cancer CSCs.

### Antagonizing Reactive Oxygen Species

Chemoradiation causes reactive oxygen species to accumulate in cells, damage DNA and kill cells. To protect DNA from endogenous or exogenous reactive oxygen species, epithelial stem cells have a strong free radical scavenging system (Diehn et al., 2009). Urothelial epithelial CSCs contain high levels of the antioxidant enzymes superoxide dismutase SOD2 and heme oxygenase, which protect the DNA of CSCs from damage by chemoradiotherapy (Schinkel and Jonker, 2003; Eisele et al., 2007). It is reasonable to speculate that the same will happen to BCSCs.

### Apoptosis Inhibition

Urinary epithelial CSCs express high level of mRNAs encoding for IL11, IL18 and IL23). Interleukins have been shown to promote the survival and growth of tumor cells *in vitro* by activating the expression of anti-apoptotic genes. These apoptotic genes include cFLIP/FLAME-1 and Bcl-xL (He et al., 2009). High level of interleukin expression is found in prostate, breast, bladder and colorectal cancers (Todaro et al., 2007). Among them, IL23 is believed to involve in inflammatory responses which preferentially activate STAT-3 pathway; STAT-3 has many tumor-promoting functions, such as promoting tumor survival, proliferation, invasion and angiogenesis. Therefore, IL23 may also play a considerable role in promoting tumor (Aggarwal et al., 2009).

In conclusion, bladder cancer CSCs express many cytokines which render CSCs better tolerance to drug therapy and mediate tumor recurrence. Therefore, investigation and validation of molecular mechanisms involved in CSCs drug resistance are of great significance to establish accurate and effective treatment options.

## Bladder Cancer CSCs Targeted Therapy

Monoclonal antibodies targeting tumor specific antigen is the most effective anticancer treatment. Analyses using immunofluorescence and fluorescence-activated cell technology confirmed that CD47 is widely expressed in most urothelial carcinoma cells, and CD44^+^ CSCs have higher CD47 expression level compared to subset of CD44^−^tumor cells. CD47 interacts with plasma membrane protein SIRPa which is mainly expressed in bone marrow cells (macrophages, neutrophils, eosinophils and dendritic cells); this results in reduced immune cell activities and thus inhibition of macrophage phagocytosis (Tripurani et al., 2011). *In vitro*, blocking CD47 using monoclonal antibodies could induce macrophage phagocytosis in muscle invasive bladder cancer cells (Chan et al., 2009). Treatment of mice transplanted with muscle invasive bladder cancer cells using monoclonal anti-CD47 antibodies significantly reduced muscle invasive bladder cancer cells in a dose-dependent manner (Willingham et al., 2012). Li et al. isolated and identified a subset of cells with stem cell characteristics (BCMab1+/CD44+ subpopulation) in which Hedgehog signaling pathway was activated and glycosylation enzyme GALNT1 was highly expressed. Bladder perfusion of GALNT1 siRNA and SHH inhibitor cyclopamine could significantly inhibit growth of bladder tumor (Li et al., 2016). BCSCs are the keys for recurrence and progression of bladder cancer. This study successfully identified a subset of bladder cancer stem cells that respond to GALNT1 siRNA and SHH inhibitors, providing a potential strategy for prevention and targeted treatment for recurrent bladder cancer.

Yang et al. found that CD44^+^ cells present in basal cell layer of normal urothelial and urothelial carcinoma (Yang and Chang, 2008). Chan et al. examined expression of CD44 in a sample contained more than 300 bladder transitional cell carcinoma tissues. The investigators found that approximately 40% of the bladder transitional cell carcinomas contained CD44^+^ cells. Subsequently, by using serial xenograft models, they identified a subpopulation of tumor cells with expression of CD44 from freshly isolated human bladder transitional cell carcinoma. These CD44^+^ cells were 10–200 times higher than those of CD44^−^cells. Later, the authors investigated heterogeneity of different CD44^+^ bladder transitional cell carcinoma subpopulations and they detected a variety of self-renewal proteins in active form, such as nuclear Bmi-1, Stat3, and p-catenin. They also found that GIi1 gene expression activity was higher in 85% of the CD44^+^ tumor cells, suggesting that the GIi1 gene may be a important molecular target (Chan et al., 2009).

CD47 may be the potential targets for cancer therapy. As mentioned above, bladder cancer CSCs may have high level of drug resistance. During the use of drugs, ways to avoid or minimize drug resistance are aspects that should not be overlooked in order to achieve therapeutic effects. On this basis, enhancement of drug specificity using bladder cancer CSCs-specific markers can significantly prevent tumor resistance. Unlike conventional radiotherapy, we expect that chemoradiotherapy and stem cell pathway small molecule inhibitors targeting CSCs will destroy tumors more accurately.

## Conclusion and Perspectives

Cancer stem cell theory was first discovered in human hematological malignancies and subsequently applied to other solid tumors. At present, experimental subjects using cells extracted from patient tumor specimens and genetically engineered mice greatly enhance our understandings on stem cell theory. However, classic stem cell theory has yet to achieve clinical success. Solid CSCs are usually isolated as single cells. However, compared to hematological malignancies, their cell formation requires cell-cell and cell-matrix interactions. Further, they largely require involvement of endothelial cells, fibroblasts and inflammatory cells in formation of tumor microenvironment; such conditions remain difficult to simulate. Regarding origin and production mechanisms of CSCs and their existence in solid tumor, there are no consensus conclusions yet and many unknowns remain to be discovered.

There are currently no effective treatment for a variety of malignant tumors including bladder tumor, and most tumor prognosis are unsatisfactory. Theory of BCSCs has been proposed in recent years. There are many questions with regard to the theory remain to be solved, and a large number of studies are required prior to clinical application. During studies of BCSCs, it is of utmost importance to identify specific CSCs markers of bladder cancer, and to isolate and identify BCSCs with self-renewal and differentiation abilities. Findings of such studies can provide better elucidation about BCSCs, and provide constructive data for classification, prognosis, treatment and early intervention of the disease. We believe that diagnosis and treatment of bladder cancer will make great progresses in the near future with more in-depth understandings of BCSCs.
